# Designing meta-resources for mathematics teachers in the context of curriculum reforms: the case of digital technology use and student autonomy in France

**DOI:** 10.1007/s11858-021-01299-2

**Published:** 2021-09-03

**Authors:** Ghislaine Gueudet, Birgit Pepin, Marie-Pierre Lebaud

**Affiliations:** 1 University of Brest, Brest, France; 2 Eindhoven University of Technology, Eindhoven, The Netherlands; 3 University Rennes 1, Rennes, France

## Abstract

The study presented in this paper concerns the design and evaluation of curriculum material that supports mathematics teachers’ understanding and enactment of reform curricula and innovative teaching practices. Our focus is on curriculum material supporting mathematics teachers’ practices combining the use of digital technology and the development of student autonomy. We refer to the theoretical framework of the Documentational Approach to Didactics, which considers teachers’ documentation work as a central lever for the evolution of teachers’ practices. Our study took place in the context of curriculum reforms in France, which called on teachers to combine the use of digital technology with the development of student autonomy in their practices. We investigate in this paper the design of a meta-resource with the aim of supporting teachers’ documentation work in this context. The documentation work ranged from choosing a lesson plan offered on a website to designing a completely new lesson. Using a design research approach, we conducted two design and evaluation cycles, involving different groups of researchers and teachers, and we analysed these design processes and their outcomes. The researchers used particular categories to distinguish between different forms of autonomy, and criteria concerning the articulation between student autonomy and digital technologies. The teachers provided elements concerning the features of a lesson plan facilitating its appropriation, and more generally related to their actual design work. Our results illustrate how a multidisciplinary team of researchers can collaborate with teachers to design ‘meta-resources’ supporting teachers’ documentation work in a context of education reform.

## Introduction

The study presented in this paper concerns the design and evaluation of curriculum material that supports mathematics teachers’ understanding and enactment of reform curricula and ‘innovative’ teaching practices. We focus on teaching practices combining the use of digital technologies and the development of student autonomy. Curriculum reforms in different countries call for the evolution of such teaching practices (see e.g., Drijvers et al., 2015), and this evolution can be challenging for teachers.

Our study took place in France, where a reform (starting in 2016) introduced a new national curriculum for secondary school. This new official curriculum emphasized in particular the development of student autonomy; it was also linked to an educational policy foregrounding the use of digital technology, in particular, a ‘digital plan for schools’^1^ announced in 2015. These new expectations raised an issue for the teachers: how could they enact this reform? Integrating digital technology in their teaching has proven to be complex (Ruthven, 2009); combining this integration and the development of student autonomy was a real challenge. Note that here, ‘digital technology’ (DT in what follows) includes a wide range of digital tools, which can be subject-specific (e.g., dynamic geometry software) or general (e.g., online collaborative writing tools). Concerning ‘curriculum materials’, we consider that they include (e-)textbooks, and also various kinds of resources designed to support teachers’ work linked with an official curriculum (e.g., digital platforms, Tamborg, 2021).

The work we present is rooted in the design research project IDEE,^2^ funded in this context (2017–2021). The aim of the IDEE project was to support teachers facing the challenge of using DT and fostering student autonomy. Several levers were developed in the project for this aim, including examples of lesson plans; professional development courses, etc. We focus here on one aspect of the IDEE project, namely, the design and evaluation of a ‘meta-resource’ for teachers (see Sect. 3.1 for the definition of meta-resource). The aim of this meta-resource was to support the enactment of the reform by teachers. The work presented here concerns the design and evaluation of an initial version of this meta-resource (prior to its use by teachers).

After a presentation of the French reform context in the second section, in the third section we introduce the theoretical elements used for this study and how we drew on related work. In the fourth section, we explain the methods used in our design study. In the fifth, we describe the first design cycle, driven by the objective of assessing the quality of lesson plans, and we present the meta-resource produced during this cycle. In the sixth, we describe the second cycle and the evaluation of the meta-resource. Finally, we discuss our results and present our conclusions.

## Context: a reform in France

In France, the Ministry of Education prescribes the national curriculum from kindergarten to the end of secondary school. This official curriculum has changed regularly since the beginning of the twenty-first century, with more reforms than during the two previous centuries (Gueudet et al., 2017). A reform introduced in September 2016 concerned primary and lower secondary school education (grades 1–9), and we focus here on lower secondary education (grades 6–9, students aged 11–15).

Our work concerns two particular aspects foregrounded by the reform. The first emphasises the inclusion of DT. Since 2016, the use of DT in mathematics classrooms has been expected to start at grade 1 (age 6), and a national ‘digital plan’ for schools helped to provide schools with tablets and Internet access. The second aspect concerns ‘student autonomy’. The official curriculum has significantly evolved since 2006, with the emergence of a common core of knowledge and skills (MEN,^3^,^2006^). This common core contains a list of knowledge aspects, competencies and attitudes that all students should acquire during compulsory education. ‘Autonomy’ was introduced as one of the seven competencies highlighted in this common core. It was defined as ‘the possibility to discuss, to act and make informed choices by developing the capacity to decide on her/his own’ (MEN, 2006, p. 23).

In 2016, the common core curriculum evolved to become the common core of ‘knowledge, skills and culture’, now structured in five areas. ‘Autonomy’ was not an area as such, but permeated the different areas, and was also presented in the introduction of the curriculum (MEN, 2015) as a major aim of teachers’ pedagogic practice: “the student should develop his/her autonomy and his/her capacity to allow him/herself thinking for him/herself” (MEN, 2015, p. 217). We note that this definition presents autonomy as an individual capacity, and does not provide teachers with directions concerning how to contribute to its development.

## Theoretical frame and related works

We introduce in this section the Documentational Approach to Didactics (DAD), the main theory framing our study. Then we present other concepts linked with our specific focus, namely, resource quality and meta-resource, student autonomy, and its links with DT.

### The documentational approach and the research question studied

DAD (e.g., Gueudet & Trouche, 2009; Trouche et al., 2019) explains and describes the processes involved when teachers interact with resources. The term ‘resource’ has a very general meaning in DAD; in this study we focused on curriculum resources (Pepin & Gueudet, 2018) and DT.

In the DAD, teachers are considered as designers of their teaching (Pepin et al., 2017). Teachers search for resources, adapt them to their own needs and aims, enact them in class, revise them, etc. We call involvement in these activities the teacher’s *documentation work*. Drawing on the instrumental approach (Rabardel, 1995), those using the DAD consider that during this documentation work, for a given goal of their professional activity, teachers develop *documents*, combining resources and professional knowledge. This process is called a *documentational genesis* (Trouche et al., 2019). Documentational geneses hold a central place in teachers’ professional development.

In terms of the DAD, supporting the enactment of a reform by teachers means fostering documentational geneses aligned with the objectives of the new curriculum. Several means can be used for this aim, such as offering ready-for-use lesson plans, organising professional development courses, etc. We focus here on a particular means, namely, designing and evaluating a tool to support teachers’ documentation work, ranging from the choice of an appropriate lesson plan on the Internet to the design from scratch of a new lesson (with the aim of integrating DT and fostering student autonomy). Within the frame of DAD, such a tool is considered a *meta-resource*: “The term *meta-resource* refers to a resource that helps to design other resources by creating a reflective posture on the documentation work to be conducted, or on its effects” (Prieur, 2016, p. 75). What makes a resource ‘meta’ is that it is designed to support teachers’ documentation work and to trigger reflection.

Using the meta-resource concept, the research question we investigate is the following:

*How can a meta-resource that supports mathematics teachers’ understanding and enactment of particular aspects of reform curricula and innovative teaching practices concerning the use of technologies and the development of student autonomy be designed and evaluated?*

This question is essential in a context where reforms are accompanied by the provision of a large number of resources from which teachers must choose, and make decisions concerning appropriation for their own teaching. It directly concerns the possible contributions of research to the implementation of reforms through the design of relevant meta-resources.

We develop further issues concerning the notion of meta-resource and related concepts in the next section.

### Supporting teachers’ documentation work

Teachers’ documentation work has multiple aspects, which range from choosing a lesson plan on a website to designing and implementing a completely new lesson. Hence, supporting this documentation work can take multiple forms, linked with these different aspects.

The choice of a resource relevant for a given teaching aim is linked to the evaluation of the ‘quality’ of this resource; this raises the complex issue of conceptualising quality. In the literature we find different definitions of *quality* (e.g., van den Akker, 2013). In the context of reform, the two criteria of ‘relevance’ (there is a need for this resource, linked with the new curriculum) and ‘practicality’ (the resource is useful/useable in practice) seemed to be important.

Nevertheless, quality also depends on the goal and purpose of the documentation work, and also on the user. With this perspective, in the frame of the Intergeo project concerning resources presenting lessons using dynamic geometry systems (DGS), Trgalová and Jahn (2013) designed a ‘quality questionnaire’ inviting the user to rate the quality of the resources (available on the Intergeo website) they used according to the following nine dimensions: (1) metadata, (2) technical aspect, (3) mathematical dimension, (4) instrumental dimension, (5) DGS added value, (6) didactical implementation, (7) pedagogical implementation, (8) integration in a teaching sequence, and (9) ergonomic aspect. Users were invited to express their satisfaction on a four-level scale: this produced an assessment linked with the users’ goals.

In the study reported by Pepin et al. (2015), we investigated the issue of the quality of e-textbooks. Considering that a re-conceptualisation of quality is necessary for e-textbooks, we coined the term ‘connectivity’ and developed a frame for assessing the connectivity of an e-textbook.

Another example is the Digital Typology created by Choppin et al. (2014). The authors outlined three categories to consider for the analysis of Digital Curriculum Resources and programs, namely, students’ learning experiences, curriculum use and adaptation, and assessment systems.

We note that all these studies provide frames that can support teachers’ choice of specific resources. Moreover, in these frames this choice is regarded as the first step in the teachers’ design of their own curriculum. Hence, we deem that investigating resources’ quality leads naturally to a meta-design perspective (Fischer, 2007), considering teachers as co-designers.

Pepin (2012) used a mathematical task analysis frame as a ‘catalytic tool’ for feedback and teacher learning. Working with teachers, Adler (2021) proposed a ‘Mathematics Teaching Framework’ (MTF), which is a structured set of principles guiding the design of their lessons. She provided evidence of the contribution of such an ideational resource (‘pertaining to ideas’) for levering educational change in the context of South Africa. The concept of meta-resource was introduced by Prieur (2016) in a similar perspective. In her study, she worked with teachers of mathematics, physics and biology within a multidisciplinary group to design a meta-resource, namely, a framework supporting the collective design of inquiry-oriented lessons. She evidenced that the involvement of the teachers in the design of a meta-resource led to an evolution of teachers’ professional knowledge about inquiry-based teaching (recommended by the educational authorities). Our study is closely related to these works, as we consider that a meta-resource can be used for assessing the quality of existing lesson plans, at the same time as a frame guiding the design of new lessons.

### Student autonomy and digital technologies

Student autonomy has been studied by many researchers in education, mathematics education in particular, using different approaches (without all referring to this terminology). Self-regulation, or self-regulated learning, is defined as the planning, monitoring, controlling, and reflection on, one’s progress toward a goal (see e.g., Zimmerman, 2000). We consider that these concepts are directly linked with student autonomy. Nevertheless, we use the term ‘autonomy’ rather than ‘self-regulated learning’, since our view on students’ activity is driven by the theory of didactical situations (Brousseau, 1997), focusing on the interactions between a student and a situation, and not on students’ goals.

Yackel and Cobb (1996) proposed distinguishing between autonomous and heteronomous students according to how they make their mathematical decisions. The autonomous students refer to their own capacities, while the heteronomous ones search for an external authority. Nevertheless, as noted by Wood (2016) for example, the importance of social aspects in autonomy has been increasingly evidenced by research, and we take these aspects into account in our study.

We first propose a distinction. Student autonomy can concern the mathematical activity: when learners analyse the mathematical tasks proposed, they choose procedures based on their perceived relevance (e.g., Pape et al., 2003). At the same time, it can be independent of the content, for example, managing the homework for the preparation of an assessment/test. We call *mathematical autonomy* the aspects specific to the mathematical content, and *transversal autonomy* the aspects independent of the subject studied.

Moreover, as stated by Ben-Zvi and Sfard (2007), the features of autonomy depend on the content studied. They distinguish between familiar and unfamiliar knowledge. In this study we consider and distinguish these two levels within mathematical autonomy: we distinguish between the mobilisation of already available knowledge, in particular procedural fluency, and the discovery of new knowledge (e.g., in problem solving). These considerations lead us to three categories: *transversal autonomy*, *autonomous mobilisation* of familiar mathematical knowledge and *autonomous development* of new mathematical knowledge.

How DT use can foster student autonomy is a broad issue, and we give here only a brief account of dimensions relevant in our study. Some kinds of DT are likely to support transversal autonomy: for example, ‘Technology-enhanced learning environments’ (TELE, Bartolomé & Steffens, 2011), in which planning tools are proposed, or digital collaboration tools fostering collective work (Faggiano et al., 2005).

Concerning mathematical autonomy, e-textbooks or digital platforms can propose interactive tasks offering relevant feedback (Rezat, 2021), which can enhance ‘autonomous mobilisation of familiar mathematical knowledge’. Specific environments or software (e.g., Dynamic Geometry Systems) can allow students to formulate and test hypotheses, and contribute to ‘autonomous development of new mathematical knowledge’. While such a use of mathematical software can foster autonomy, it can also require a particular autonomy for relevant use of this software.

## The study

In this study we used a Design Research (DR) approach (van den Akker, 2013; Bakker, 2018; Cobb et al., 2003) to develop and evaluate a ‘meta-resource’ for helping (mathematics) teachers to respond to the French curriculum reforms regarding student autonomy and the use of DT. The present study had two cycles, as follows:

1. [Cycle 1] A design and formative evaluation cycle, where the criteria for the meta-resource were developed from the literature and formatively evaluated by the design teams (see below, groups 1–4);
2. [Cycle 2] A design, enactment and evaluation cycle, where the designed meta-resource was trialled out (in lesson planning), enacted and formatively evaluated by the participating teachers.

In the following we first present the general collective organisation for designing the meta-resource, and secondly, the data collected and how we analysed the data.

### Participants and organisation of the design teams

The design of the meta-resource was part of the IDEE project; as such, different groups contributed to this design. In the table below (Table 1) we present the groups related to mathematics. The group labelled (n + 1) is always a subgroup of group n.

**Table 1:** The different groups, their function and their organisation

Group	Members	Function	Organisation
Group 1—IDEE	50 members: researchers in education (mathematics, science, English; DT use), researchers in sociology and economy, PhD students, teacher educators, representatives of the local educational authority, teachers	Discussing theoretical issues Sharing and discussing the work of the subgroups (including the design of the meta-resource)	2 or 3 meetings (3 h long) each academic year
Group 2—didactics group	20 members: members of IDEE working in mathematics, science, and English education	Discussing theoretical issues Sharing and discussing the work of the subgroups for each school subject (including the design of the meta-resource)	6 meetings (3 h long) each academic year
Group 3—mathematics group	10 members: 4 researchers and teacher educators, and 6 lower secondary school mathematics teachers	Design and trial of lesson plans Design and trial of the meta-resource	Worked from September 2017 to June 2019. 6 meetings (3 h long) each academic year
Group 4—mathematics education research group	4 members: researchers and teacher educators	Review of the literature Design of the meta-resource	Distant work, asynchronous

All these groups had shared folders (one for each group) on a distant platform. We followed the work of these groups from January 2017 to September 2019. The first and third authors of this paper were also members of these groups. As such, they participated in all meetings of the four groups. The mathematics teachers in group 3 can be considered as expert teachers. They had between 5 and 25 years of experience as teachers, and all of them had already participated in design and research groups with mathematics education researchers.

### Data collection and analysis

Design cycle 1 took place from January 2017 to July 2018. An initial version of the meta-resource was created; then it evolved during its trial for the assessment of existing lesson plans, and according to discussions in the different groups. The data collected about this cycle are ‘natural data’, namely, files produced during the work of the four groups. We collected these files on the platform that was used to share them. They included the following:

- The written reports of each meeting: 4 reports for group 1, 11 for group 2 and 6 for group 3 (concerning these meetings we also had personal notes).
- The different versions of the meta-resource. Group 4 was responsible for producing these versions, incorporating the outcomes of all the discussions during the meetings of groups 1–3. We considered 6 successive versions of the meta-resource produced during cycle 1.

For analysing these data, we started with the different versions of the meta-resource and noted each time the evolutions between the two versions. Then we identified the sources of the evolution in our notes or in the meeting reports. We noted in particular in which group the evolution was suggested, and the arguments supporting this evolution.

Design cycle 2 took place from September 2018 to September 2019. During this design cycle, in the ‘mathematics group’ (group 3) several lessons were designed and tested in class. The meta-resource was used as a guideline for the design of these new lessons. We have chosen here to focus on a lesson on probabilities for grade 9. Several versions of this lesson were enacted in class. We observed and video-recorded one of these enactments of the lesson (organised in January 2019). The teacher was Sophie, one of the members of group 3. The choice of Sophie (amongst the six teachers in group 3) was the consequence of organisation issues (possibility for the researchers to be present). The implementation in her grade 9 class corresponded to five sessions of approximately 50 min each. The first four sessions were video recorded and observed by a researcher on our team. The students’ productions (on paper or on the computers) were collected, and Sophie described her impressions concerning the work during meetings of group 3.

We analysed the sessions in class in terms of student use of DT and autonomy. This was done, first, by observing how the class was organised: for example, there were teacher interactions with the whole class, group work (e.g., whether, during group work, the groups called for the teacher or only talked with her when she came by). Second, we investigated how the students reached the mathematical objectives of each step (see Appendix) through their productions, the direct observations and Sophie’s accounts. The aim of this analysis of the actual students’ activities was to contribute to the evaluation of the meta-resource. Indeed, the meta-resource was used as guide in the design of the lesson. Sophie was a member of the designers’ group, and her enactment of the lesson corresponded as far as possible to the lesson intended by the group. Thus, the analysis of the students’ activity should reveal whether or not there were discrepancies between the intentions of the lesson as identified by the meta-resource and the actual enactment in terms of student autonomy. Very limited discrepancy would mean that the meta-resource has been useful.

## Findings from design and (formative) evaluation cycle 1

**Fig. 1:**
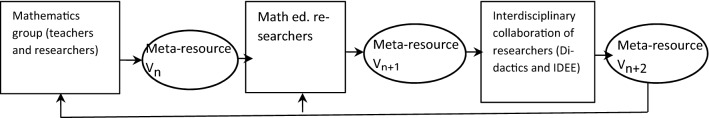
Representation of the first design cycle (V_n_ for the nth version)

In this section we analyse the first design (and evaluation) cycle of the meta-resource and we present the design criteria and the version of the meta-resource resulting from this cycle. We investigated three major aspects of this design. This analysis did not follow a chronological order; indeed, some of these processes were parallel, and many local adjustments of the meta-resource occurred. The figure below provides a representation of this cycle (Fig. 1).

### Collaboration of mathematics education researchers and mathematics teachers (group 3)

For this step of the design of the meta-resource in the mathematics group (group 3), the mathematics education researchers (group 4) developed an initial version of the meta-resource from the literature, which was then presented to, and formatively evaluated by, the teachers. The aim of this initial version was to assess the quality of existing lesson plans and/or to redesign them (in terms of DT use and student autonomy).

A central source of inspiration for Group 4 was the Intergeo quality questionnaire (see Sect. 3.2; Trgalová & Jahn, 2013). We chose a similar structure, namely, some general categories, each of them corresponding to a list of more detailed criteria. These criteria were rated on a four-level scale, from 0 (not present in this lesson plan) to 3 (very good). A letter rating the category was allocated drawing on the marks attributed to each item, as follows: (A) for more than 75% of the maximum, (B) for between 50 and 75%, etc. This system produced a synthetic profile of the lesson plan.

Concerning the categories, in previous research work in which we designed meta-resources for assessing the quality of lesson plans for specific purposes (e.g., Besnier et al., 2014), we noticed that choosing nine categories was not practical, and hence we proposed to reduce their number to five. The five categories retained were as follows:

1. The lesson plan is clear and complete (it provides all the elements needed for implementing the lesson).
2. The lesson plan is easy to adapt for the specific context of the user and to implement.
3. The mathematical content of the lesson is rich (e.g., in terms of mathematical autonomy).
4. The use of DT is relevant (e.g., supports mathematical autonomy).
5. The lesson can foster transversal autonomy.

For each category we (the researchers) wrote a first list of criteria (from 4 to 8 criteria), again drawing on theory and previous research. The Intergeo quality questionnaire was the central source of inspiration for category 1. The documentational approach was the central inspiration for category 2. The criteria in categories 3, 4 and 5 came from our literature review concerning student autonomy and DT (Sect. 3.3). This first version of the meta-resource was submitted to mathematics teachers (within group 3). The teachers subsequently suggested new criteria. Concerning the use of DT, they emphasised that the lesson should not require Internet access. Indeed, in many schools in France, stable access to the Internet is not guaranteed. They also suggested adding something like ‘possibilities of adaptation of the software to students’ needs’. This suggestion was discussed, because it seemed to be linked with specific software, and the meta-resource should apply to various kinds of DT. It was finally rephrased as: ‘The use of DT allows the teacher to take into account the diversity of students, for example by providing for personalised learning paths’. Summarising this aspect of the design process, we could say that it combined several sources:

- External sources, coming from the research literature—theories and research results about quality assessment and about autonomy;
- Internal sources, experience of the participants—the experiences of the researchers involved, in terms of meta-resources for teachers, and teachers’ experience in terms of DT use and student autonomy.

### Formative evaluation of the meta-resource: assessing lesson plans (group 4—mathematics education researchers)

After producing an initial version of the meta-resource, the members of group 4 trialled it by assessing lesson plans available on the web; such a trial step was reiterated after each significant change in the criteria. The organisation of this trial was as follows. We selected lesson plans designed by teachers and available on an online institutional platform called ‘Cartoun’.^4^ This platform was created in 2016 to support DT use in class. We selected eight lesson plans pertaining to mathematics at lower secondary level and using various DTs, for example, dynamic geometry, interactive exercises, videos, and Scratch.

**Fig. 2:**
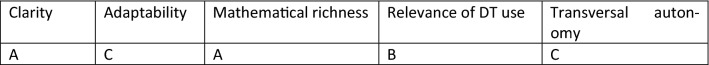
Example of a final profile obtained by analysing a lesson plan with the meta-resource

Each lesson plan was coded, using the meta-resource, by two researchers on our team. Our aim was not to obtain exactly the same coding for each criterion, but that the final profile produced was the same (see an example in Fig. 2).

For example, the profile in Fig. 2 characterised a lesson plan that was clearly presented, but not easy to adapt (e.g., the students’ sheets were presented only as PDF files). It was mathematically rich (i.e., based in a rich problem), but the DT use could be improved and the possibilities of transversal autonomy of students were limited (e.g., no help was planned).

The modifications resulting from this step were twofold. First, we observed that some lessons could not be taught without DT: indeed, the reform introduced Scratch as a part of mathematics in the official curriculum. Hence, we again introduced two cases with different criteria, namely, for a lesson that could be taught without DT (e.g., in geometry) and for a lesson that could not be taught without DT (for the topic of Scratch). The second modification consisted in considering some of the criteria as ‘bonus’: for these criteria the mark proposed could lead to improving the final letter attributed to the category (e.g., from B to A) but not to lowering it. For example, the criteria concerning students’ collective work were placed as bonus: collective work can foster autonomy, but individual work can be relevant as well.

Moreover, the differences in coding among different researchers were discussed, and led to changes in the formulation of criteria, in order to avoid as far as possible different interpretations.

In this aspect of the design, we identified two central processes. One was the clarification and stabilisation of the criteria resulting from a collective work of mathematics education researchers, a kind of ‘saturation’ process. Moreover, testing the meta-resource on lesson plans revealed the need for distinguishing two cases. This need had not been identified in previous work, since it was directly linked to the reform introducing algorithmics as a part of mathematics in the official curriculum.

### Collective work with the interdisciplinary research team (groups 1 and 2)

The processes presented and analysed above led to the design of a meta-resource for assessing the potential of lesson plans in mathematics (in terms of DT use and student autonomy). Simultaneously, similar meta-resources were designed for English as a second language and for physics. The three resulting meta-resources were discussed and confronted in the group of didactics researchers (group 2). The mathematics education researchers had more experience in the design of such meta-resources (based on previous work cited above). Thus, the collective work in group 2 led to significant modifications in the physics and English meta-resources, and only minor changes in the meta-resource for mathematics.

The most important modifications came from sociologists, who were asked to check the equity aspects in the meta-resource and to add criteria in the different categories if needed. These colleagues added new criteria coming from their research experience, in particular from a previous project concerning equity and the use of DT (Le Mentec & Plantard, 2015). We emphasise here two examples of such changes. First, previous research in sociology evidenced that school students were confronted with many texts, and this created difficulties for students coming from underprivileged backgrounds (Lahire, 1993). Thus, the sociologists added the need to make use of different media, in particular videos, in the help provided to students. Second, concerning the use of DT, they added a criterion about the possibility of working on a smartphone if homework was planned in the lesson. This was linked to issues of access (Forgasz et al., 2010): students from underprivileged backgrounds were expected to be equipped with smartphones rather than with computers.

In Table 2, we present the version of the meta-resource resulting from this cycle (established in June 2018).

**Table 2:** Content of the meta-resource at the end of cycle 1 (June 2018)

**1. The lesson plan is clear and complete**		**2. The lesson plan is easy to adapt and to implement**		
The activity is clearly situated within the official curriculum		The lesson plan is complete but synthetic		
The mathematical objectives are clearly stated		Elements of the lesson plan can be modified		
The contribution of digital means is clear		The use of the lesson plan is intuitive (e.g., the use of software)		
The classroom organisation is clear		Advice for adapting the lesson plan to a particular context is provided		
The mathematical prerequisites are clearly presented		Implementing the lesson does not require too much material: no Internet access in particular		
The digital prerequisites are clearly presented		Implementing the lesson does not require a very long preparation time		
Differentiation and support means are planned		Access to the files is easy enough. When software is used, it is open software easy to download and install		
Reports of classroom uses are available				
Extracts of students’ work are proposed				
Synthesis, institutionalisation and assessment means are planned				
**3. The mathematical content of the activity is rich**				
3.1 Problem-solving			3.2 Procedural fluency	
The students can take mathematical initiatives (bonus)			The students can practice techniques (bonus)	
The students can experiment, test, make conjectures				
The students can use different procedures				
The mathematical content corresponds to the announced objectives and prerequisites				
The productions required from students permit access to their reasoning process				
The activity leads to work on some of the competencies^a^: Searching/Reasoning/Modelling/Representing/Computing/Communicating				
The activity leads to using different representations and changes of representations				
Self-assessment and/or formative assessment elements are proposed				
**4. The use of digital technologies is relevant**				**5. The activity supports transversal autonomy**
4.1 The lesson could be organised without DTs	4.2 The lesson could NOT be organised without DTs			Students can work at their own pace
				Some help is planned, in case of difficulty
DTs allow the teacher to propose specific representations and/or information				Different kinds of help are available: text, video, pictures, etc.
				The students can take initiatives in the organisation of their work
DTs allow the students to have access to specific representations and/or information				The students can know if their production is correct without calling the teacher
				Tools for self-assessment are provided
DTs bring some other added value for reaching the objective (e.g., possibilities of computation)				A tutorial is provided for the software used
				Students’ collective work is proposed (bonus)
DTs permit users to make several attempts and to test their validity				The students can follow a personalised path (bonus)
DTs allow the teacher to access students’ work and show it to the class				
DTs allow differentiation, proposing different trajectories for different students				
If homework is required with DTs, it can be done with a smartphone				
DTs permit work in different places (bonus)				
DTs permit students’ mathematical collective work (bonus)				

^a^Official competencies of the French Curriculum.

## Findings of (design and) formative evaluation cycle 2

**Fig. 3:**
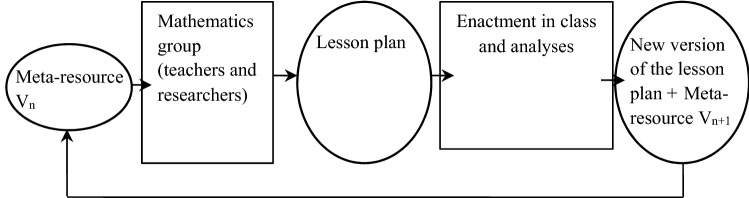
Representation of the second design cycle

In cycle 2 (Fig. 3) we initiated a new use of the meta-resource for designing and evaluating new lesson plans. The lesson plans designed were trialled in class by the teachers from group 3. These trials led to new evolutions of the meta-resource.

This work started (in September 2018) with the version of the meta-resource presented above. A first major change occurred. Teachers, who were now placed in the position of authors of the lesson plans, rejected the use of marks to produce a profile of the lesson plan with five letters (see Fig. 2 above). They considered that this felt like an evaluation of them as teachers (as they were the designers of the lessons). Thus, the evaluation with marks and letters was suppressed (and the ‘bonus’ also, as a consequence). The meta-resource was then used for providing guidelines for the lesson design by checking it against the criteria.

In this section we present and analyse an iteration of cycle 2 linked with a lesson about the introduction of probability in grade 9. First, we examine the lesson plan designed by group 3 and study whether or not it respected the criteria (Sect. 6.1). Second (Sect. 6.2), we examine the trial of the lesson in class, informing us about the link between these criteria and the actual autonomy of students in class. Note that we do not study how the meta-resource was used by group 3 to produce the lesson plan, but how the design and enactment of this lesson intervened in the process of building/improving the meta-resource.

### Design of a lesson (on probability) and relevance of the meta-resource

**Fig. 4:**
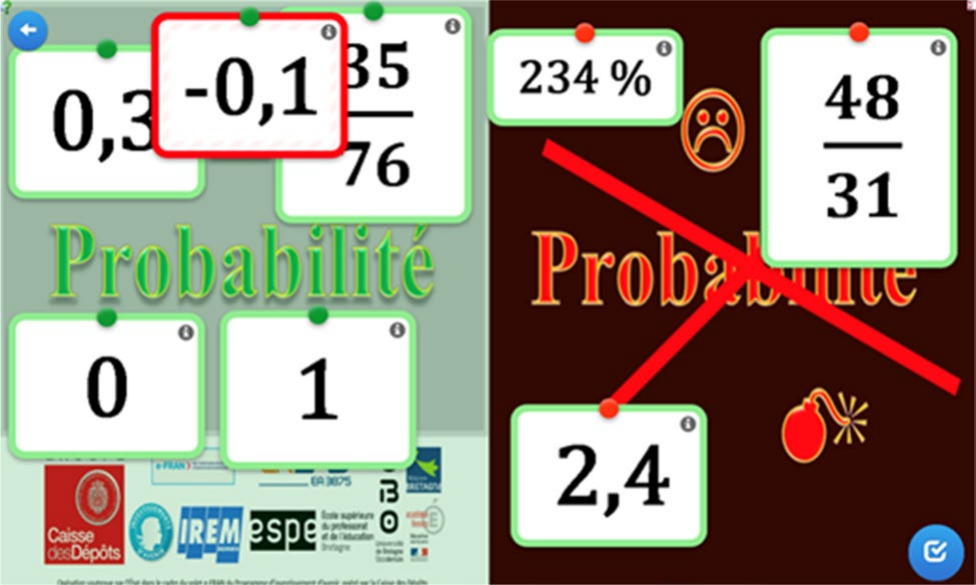
Example of an interactive exercise: *Could this value be a probability or not?*

Mathematics teachers involved in the ‘Mathematics group’ chose to focus on the teaching of probabilities at grade 9. They designed a lesson plan with four successive steps. Firstly, the students played in groups of four (two pairs playing against each other) a game called ‘Conquest’.^5^ Its aim is to attain, by the sum of the numbers on two die faces, the numbers from 1 to 12. This game highlighted that such numbers (sum of those on two dice) do not have the same probability of being obtained (for instance, a sum of 1 will never appear). The vocabulary of probabilities was introduced by the teacher, and the students had to watch a video at home that recalls this vocabulary. Next, they had to write (still in teams of four) on a paper a mind map about probabilities, where the concepts (sample space, event, etc.) were networked and illustrated by examples coming from the Conquest game. Then they worked on interactive exercises (Fig. 4).

The teacher presented a synthesis of all the notions and properties encountered. Finally, the students worked on computers (in pairs) and programmed a simulation of the experiment ‘sum of two dice’ with a spreadsheet or with Scratch (or both if there was enough time for this).

The complete lesson plan is presented in the Appendix. Here we comment on specific aspects of it, following categories 3, 4 and 5 of the meta-resource.

- *Richness of the mathematical content (category 3, here 3.1):* The teachers knew the game Conquest from their previous experience in research groups, and chose it to develop students' mathematical autonomy. They designed online interactive exercises to offer possibilities for self-assessment.
- *Relevance of DT use (category 4, here 4.1*): the spreadsheets or Scratch seemed relevant to this topic of probabilities, since they allowed the simulation of a large number of random experiments. The interactive exercises permitted students to make several attempts and to test their validity. However, we proposed a paper version of these exercises for teachers who had no Internet access; the objective of the lesson can be reached without interactive exercises. One criterion in category 4 was not met: indeed, the students’ productions did not allow the teacher to access student reasoning as expected.
- *Supporting transversal autonomy (category 5*): The teachers chose to enhance the possibility for students to work at their own pace and to receive feedback, without calling the teacher. For this reason, they designed the interactive exercises. They chose also to develop students’ collective work, discussing introductory questions with the whole class and organising regular pooling.

The meta-resource was not systematically used during the design of the lesson plan: the members of Group 3 knew about its categories, and checked only from time to time if their lesson plan took all aspects into account. The comments above reveal that the application of the meta-resource to this lesson plan evidences some possibilities for improvement; nevertheless, it led to concluding that the potential of this lesson in terms of DT use and student autonomy was high. We now compare this a priori estimated potential and the actual DT use and student autonomy during the implementation of the lesson.

### Implementation of the lesson and further evaluation of the meta-resource

In this section we analyse student autonomy and DT use during the lesson that was enacted by Sophie.

#### Mathematical autonomy

Concerning probabilities, the Conquest game contributed to student mathematical autonomy. At the end of the game, Sophie asked the students what they retained from it. One student said ‘you need to be lucky!’ then several answered ‘not only!’, in particular with one student saying: ‘It is not a matter of luck, but also of computing. For example, you should not play 12, because there is only one way to obtain 12’.

Concerning programming with Scratch (belonging to mathematics, according to the French official curriculum), the students in this class already knew this software. Sophie handed them an incomplete program on Scratch, and they successfully completed the blanks in this program. We note nevertheless that in the lesson designed by our group, the incomplete program was given to students on a sheet of paper and they had to implement it themselves on Scratch. Thus, this trial did not allow us to make conclusions concerning the possible autonomy of students with Scratch in this lesson.

#### Use of DTs related to autonomy

The use of DTs by the students took place mainly during sessions 3 and 4. The amount of work by pairs on a computer during sessions 3 and 4 was very significant (from 55 to 98% of the time). The work on interactive exercises allowed students to work at their own pace. During our observation, we saw that different pairs were working on different exercises at the same moment. The feedback on the exercises also helped the students to adjust their answers. Scratch also contributed to their autonomy. Indeed, after completing the program, the students used it to simulate a great number of trials of this random experiment, and noted the frequency of each outcome from 2 to 12.

#### Transversal autonomy

Students worked collectively, either in groups of four, or in pairs, for more than 65% of the time during the four sessions observed. This collective work fostered students’ transversal autonomy. Sophie always walked around the class and talked with the groups during the lesson. The students discovered by themselves the rules of Conquest; they talked with the teacher when she passed by, but did not call her. They themselves shared the responsibilities in the groups and in the pairs, listened to the arguments of other members in the group and so on. Sometimes they stood up to ask a student in another group.

The homework suggested (watch a video and cut labels for the vocabulary table) was done by only a minority of students. The students who did not watch the video evoked an access issue—but they all had smartphones and it was possible to use them for watching this video. This led to an evolution in the lesson plan after this trial, of adding the possibility of watching the video in class.

The analysis summarised above confirms that the meta-resource can contribute to the design of lessons combining the use of DT and student autonomy. Indeed, the lesson implemented by Sophie followed very closely the lesson plan written by the group, using the meta-resource. Her students’ activity actually matched the intentions of our group (group 3), when designing the lesson plan.

We also noted that some aspects of the lesson plan could still be improved to take into account possible differences in context in various classrooms. For example, for the teachers in our team, tutorials on Scratch and on the spreadsheet were not needed, since their students were already familiar with this software. With such tutorials, the lesson plan would be more usable for other teachers.

## Discussion

The research question we posed was the following:

*How can a meta-resource that supports mathematics teachers’ understanding and enactment of particular aspects of reform curricula and innovative teaching practices concerning the use of technologies and the development of student autonomy be designed and evaluated?*

The analysis of the two design cycles presented above evidenced that meaningful, collective work in the different groups helped to design and formatively evaluate (in successive cycles) the meta-resource.

The contribution of the researchers in the team was, amongst other aspects, the theory. This helped to conceptualise (and subsequently to operationalise) and collaboratively to make sense of the objectives of the reform: the national guidelines presented ‘autonomy’ as an ‘individual capability’. The use of DT was not related to student autonomy in official texts. Drawing on the literature, the researchers proposed productive categories concerning autonomy, which played an important role for the design of the meta-resource. Concerning DT, research results contributed to the proposition of criteria for category 4 (‘the use of DT is relevant’) of the meta-resource. In these criteria, the link between DT use and students’ mathematical autonomy was central. This meta-resource brings a coherence that was not evidenced in the official texts, and should thus contribute to support teachers in establishing links between different elements of the reform.

Amongst the contributions of the teachers, we note that they raised the awareness of the team in terms of the actual working conditions and practicalities in schools: the equipment in terms of computers and Internet access did not always permit the development of DT use promoted by the reform. We further note that, whilst the researchers intended to develop a meta-resource supporting different aspects of the teachers’ documentation work—evaluating an existing lesson plan (attributing grades to the different categories) or designing a new lesson plan—the teachers preferred this second type of use of the meta-resource (and rejected the grading of their own lesson plans). This coincides with the features of productive meta-resources evidenced in previous studies (Prieur, 2016): a meta-resource has to support teachers’ design of lessons (and not assess it).

The study presented in this paper clearly has some limitations. The meta-resource supported the design of a lesson plan by our group, corresponding to the expectations of the reform. However, we did not research its appropriation by teachers who were not involved in its design. This is a limitation of our study, and an important direction for future research. Another limitation is that the design of the meta-resource took place before the COVID-19 crisis; thus, it did not take-into-account the strong impact of the crisis in terms of DT use and student autonomy (Clark-Wilson et al., 2020). Nevertheless, we claim that this study conducted in France has contributions both for research and for practice internationally; we present these in the conclusions.

## Conclusions

In terms of empirical results, the present study contributes to our understanding of the collaboration between researchers in a multi-disciplinary team, and between teachers and researchers, in the context of educational reforms. The collaboration of researchers, experts in different domains, was needed in order to consider different aspects of the reform and to identify categories organising the meta-resource, and criteria in these categories. The teachers in our group added criteria coming from their professional experience, and suggested features more acceptable for other teachers (in terms of vocabulary used, but also by rejecting the grading of lesson plans). All this design work was associated with a critical stance: for example, the researchers proposed a productive characterisation of student autonomy, and of how DT use can contribute to its development. The teachers warned that the equipment in schools did not always meet the demands of DT use. Finally, our group designed a meta-resource likely to deepen mathematics teachers’ understanding of the renewed curriculum and to support their documentation work in this context.

In terms of theory, we claim that the meta-resource concept requires further attention. In design-based research, many frames have been developed to support teacher design. Confronting these frames with the concept of meta-resource as introduced in DAD could lead to interesting theoretical evolutions. Moreover, further studies about meta-resources and about their productive features, in particular in a context of reforms, are needed.

The study more generally illustrates the potential contribution of a design research approach in terms of linking theory and practice in a context of reforms. These activities of collaborative curriculum design (accompanied by appropriate research approaches) are likely to bridge the theory–practice gap, and benefit both, as follows: practically, teachers in their lesson planning and development of teachers as designers of their (own) curriculum; and theoretically, researchers in their knowledge development about teacher documentation work and about support for teacher professionalisation, in particular in view of curricular reforms. We note that all these contributions for research are internationally relevant and not limited to the context of our study.

In terms of implications for practice, the meta-resource is likely to be beneficial for teachers who want to design their own curriculum and align it with the reform efforts concerning the use of DT and student autonomy. This enterprise concerns the reform in France, but these characteristics are also promoted by the educational authorities in many countries (see, e.g., Drijvers et al., 2015). Hence, we claim that these implications for teaching also have international relevance. We have used the meta-resource in pre-service teacher education programs (Gueudet & Joffredo-Lebrun, 2021) and received very positive feedback about its usefulness from pre-service teachers who had to collectively design a lesson combining DT use and student autonomy. In our ongoing work, we further investigate issues of sustainability and dissemination of the meta-resource in teacher education programs.

## Appendix: Introducing probability at grade 9, short version of the lesson plan

Detailed lesson plan (in French) available at https://www.interactik.fr/portail/web/se-documenter/cerad-sequence-probabilite.

**Table:**

Steps	Students’ activity and resources	Classroom organisation
S0	Short presentation of the game ‘Conquest’	Whole class, end of another lesson
S1	Presentation of the lesson on probability with different possible learning trajectories Quick interactive exercises about an intuitive approach of probability Game ‘Conquest’ Synthesis of the observations on the game. First approach of the vocabulary Homework: watch the video (https://education.francetv.fr/matiere/mathematiques/sixieme/video/petits-contes-mathematiques-les-probabilites) and fill in a report, cut labels with the name of the concepts	Whole class, then group work (4 students)
S2	If needed, watch the video in class Fill in vocabulary sheets (tables or mental card), using labels with the names of the concepts and adding examples from the game Synthesis on the board by the teacher, links with the game	Individual work, then whole class
S3	Work on LearningApps interactive exercises If there is no possible Internet access, the work can be done on paper Synthesis with reference to the completed vocabulary table (or mental card)	Individual work, or by pairs on computers
S4	Simulation throwing two dice (spreadsheet and/or Scratch)	By pairs on computers

## Footnotes

1 https://www.gouvernement.fr/action/l-ecole-numerique.

2 Interactions Digitales pour l’Enseignement et l’Education (Digital Interactions for Teaching and Education). This research program is supported by the French government as part of the e-FRAN component of the Future Investment Program, operated by Caisse des Dépôts.

3 MEN stands for ‘Ministère de l’Éducation Nationale’.

4 https://cartoun.education.fr/portail.

5 Created by another research group in IREM Rennes: https://irem.univ-rennes1.fr/groupe-jeux-et-mathematiques.
